# Repeatability and validity of a standardised maximal step-up test for leg function-a diagnostic accuracy study

**DOI:** 10.1186/1471-2474-12-191

**Published:** 2011-08-19

**Authors:** Lillemor A Nyberg, Mai-Lis Hellénius, Jan Kowalski, Per Wändell, Peter Andersson, Carl Johan Sundberg

**Affiliations:** 1 Center for Family and Community Medicine, Department of Neurobiology, Care Sciences and Society, Karolinska Institutet, Stockholm, Sweden; 2 Karolina Primary Health Care Center, Karlskoga, Örebro County Council, Sweden; 3 Department of Medicine, Karolinska Institutet, Stockholm, Sweden; 4 Department of Clinical Science, Intervention and Technology, Karolinska Institutet, Stockholm, Sweden; 5 Department of Physical Therapy and Department of Orthopedics, Karlskoga Hospital, Karlskoga, Örebro County Council, Sweden; 6 Department of Physiology & Pharmacology, Karolinska Institutet, Stockholm, Sweden

## Abstract

**Background:**

Objectively assessed physical performance is a strong predictor for morbidity and premature death and there is an increasing interest in the role of sarcopenia in many chronic diseases. There is a need for robust and valid functional tests in clinical practice. Therefore, the repeatability and validity of a newly developed maximal step up test (MST) was assessed.

**Methods:**

The MST, assessing maximal step-up height (MSH) in 3-cm increments, was evaluated in 60 healthy middle-aged subjects, 30 women and 30 men. The repeatability of MSH and the correlation between MSH and isokinetic knee extension peak torque (IKEPT), self-reported physical function (SF-36, PF), patient demographics and self-reported physical activity were investigated.

**Results:**

The repeatability between occasions and between testers was 6 cm. MSH (range 12-45 cm) was significantly correlated to IKEPT, (*r *= 0.68, *P *< 0.001), SF-36 PF score, (*r *= 0.29, *P *= 0.03), sex, age, weight and BMI. The results also show that MSH above 32 cm discriminates subjects in our study with no limitation in self-reported physical function.

**Conclusions:**

The standardised MST is considered a reliable leg function test for clinical practice. The MSH was related to knee extension strength and self-reported physical function. The precision of the MST for identification of limitations in physical function needs further investigation.

## Background

Primary health care handles patients of all ages for whom increased physical activity and improved physical function would be valuable to prevent and treat common chronic diseases [1-4]. Objectively assessed physical performance-e.g. low maximal oxygen uptake [5-7] or low muscle strength [8,9]-is a strong predictor of morbidity and premature death independent of physical activity and muscle mass. Interestingly, in men < 60 years the rate of loss of strength has been shown to be more important than actual strength [10]. Muscle strength provides a better estimate of mortality risk than does muscle quantity [11]. Recent evidence-based recommendations include endurance as well as resistance training [3] for health benefits. Loss of muscle mass and knee extensor strength correlates with an increased risk of falling and loss of functional independence, and has been identified as the most important factor limiting the ability to rise from a chair [12]. Finally, quadriceps muscle weakness is a primary risk factor for knee pain, disability, and progression of joint damage in persons with osteoarthritis (OA) of the knee [13], one of the most common medical conditions from midlife onwards [13,14].

One way of assessing a patient's leg muscle strength is to gauge the ability to step up. Leg function has been tested by having the patient mount an ordinary platform with five levels: step height correlated to muscle strength in older people [15,16] and patients with knee and hip OA [17]. In contrast, assessment of middle-aged meniscectomised patients based on a footstool climbing test with few levels did not provide acceptable results when test-retest and floor/ceiling effects were taken into account [18]. Although, there are several tests assessing leg function both multifactor and functional ability assessment tools [19,20], a systematic review concluded that no single tool can be recommended for implementation in all settings or for all subpopulations within each setting [21]. Many tools for the assessment of fall risk prevention among older adults have been previously studied. We sought to evaluate an assessment tool suitable for all ages by being differentiating and difficult enough to perform also for young and middle-aged persons. The test for leg strength and leg function in this study was developed in order to be a standardised procedure suitable for clinical daily use.

Standardisation, done in the first author's clinical practice, started with assessing patients' ability to step up on one or two ordinary stairway steps. This only discriminated for 18 and 36 cm. Therefore a maximal step-up test (MST) with 3-cm intervals between step heights was developed for assessment of maximal step-up height (MSH). The rationale for choosing 3-cm intervals was: 1. to have enough levels for discrimination between subjects/patients and test occasions, 2. to have sufficiently few levels not to exhaust subjects/patients and 3. to be able to come as close as possible to the highest individual MSH while taking 1 and 2 into consideration. In our study the highest step height reached with the standardised MST is denoted the "maximal step-up height" (MSH).

Accordingly, the overall aims were to evaluate a new method for measurement of lower-extremity function, the standardised MST, by assessing: 1. MSH repeatability, 2. MSH relation to isokinetic knee extension peak torque (IKEPT), health-related quality of life (SF-36, subscales for physical function (PF), bodily pain (BP) and general health (GH)), individual characteristics and self-reported physical activity (the International Physical Activity Questionnaires, IPAQ, short form) and 3. MSH cut-off for limitations in physical function (SF-36, PF).

## Methods

### Subjects

The study was performed at Karolina Primary Health Care Center in Karlskoga (Örebro County Council, Sweden), a town with 30 000 inhabitants in a rural district. Inclusion criteria were: 1) female or male, 2) 30-65 years old, 3) no self-reported complaints associated with the legs or hips leading to a visit to a health care unit in the previous six months, 4) no major health problems that interfered with climbing stairs, 5) from inactive to regularly physically active, 6) never before having been tested with a step-up test, climbing test or on an isokinetic device, 7) well motivated, and 8) understanding Swedish. Invitations were distributed to workplaces, a men's running group and their wives. Thirty women and 30 men with a median (range) age of 53 (34-63) and 55 (36-64) years, respectively (table 1) were enrolled. The female and male subjects came from about 20 different work-places, respectively: They represented a wide variety of professions and educational backgrounds. Written informed consent was obtained from all participants after oral and written information was provided. The local ethics committee at Örebro County Council approved all procedures.

**Table 1:** Descriptive statistics of subjects for age, anthropometry, self-reported energy expenditure and scores on the health-related quality of life scale SF-36, by sex

	Women, n = 30	Men, n = 30	Total, n = 60
Descriptive statistics at baseline	Mean (SD)	Mean (SD)	Mean (SD)
Age (years)	52.6 (7.0)	55.3 (6.2)	54.0 (6.7)
Weight (kg)	71.9 (11.2)	86.1 (11.3)	79.0 (13.2)
Height (cm)	166.1 (4.8)	177.9 (5.4)	172.0 (7.8)
Body mass index, BMI, (kg· m^-2^)	26.1 (4.0)	27.3 (4.2)	26.7 (4.1)
Energy expenditure, (kcal·wk^-1^)	925 (722)	1469 (1249)	1123 (344)
PF, Physical function	91.0 (10.6)	96.3 (4.5)	93.6 (8.5)
BP, Bodily pain	76.6 (24.2)	84.0 (20.0)	80.3 (22.3)
GH, General health	79.1 (20.4)	79.5 (18.0)	79.3 (19.1)

### Design

Subjects were invited in groups of ten on two test occasions (occ 1 and occ 2), one week between occasions, for intra-examiner assessment of MSH test-retest repeatability. Four weeks after occ 1, the participants came individually to the third test occasion (occ 3) for assessment of IKEPT and MSH test-retest repeatability (comparing occ 1 and occ 3). At occ 1 and occ 2, MSH was assessed twice at least 30 minutes apart, by two examiners-the first author and a tester accustomed to the MST-in random order for assessment of inter-examiner test-retest repeatability. MSH of both legs was assessed with the MST on all three occasions. The MST values remained secret between examiners and occasions, and the subjects agreed not to inform the tester of earlier results. Weight and height were measured at occ 1, and weight at occ 3. The SF-36 was assessed at occ 1. Physical activity level during the week between occ 1 and occ 2 and the week before occ 3 was assessed. The subjects were instructed to maintain their ordinary physical activity habits during the study. At occ 3 all subjects began with a MST conducted by the first author. Thirty minutes later, IKEPT was measured by a physiotherapist well acquainted with the procedure.

### Measurements and equipment

**Figure 1:**
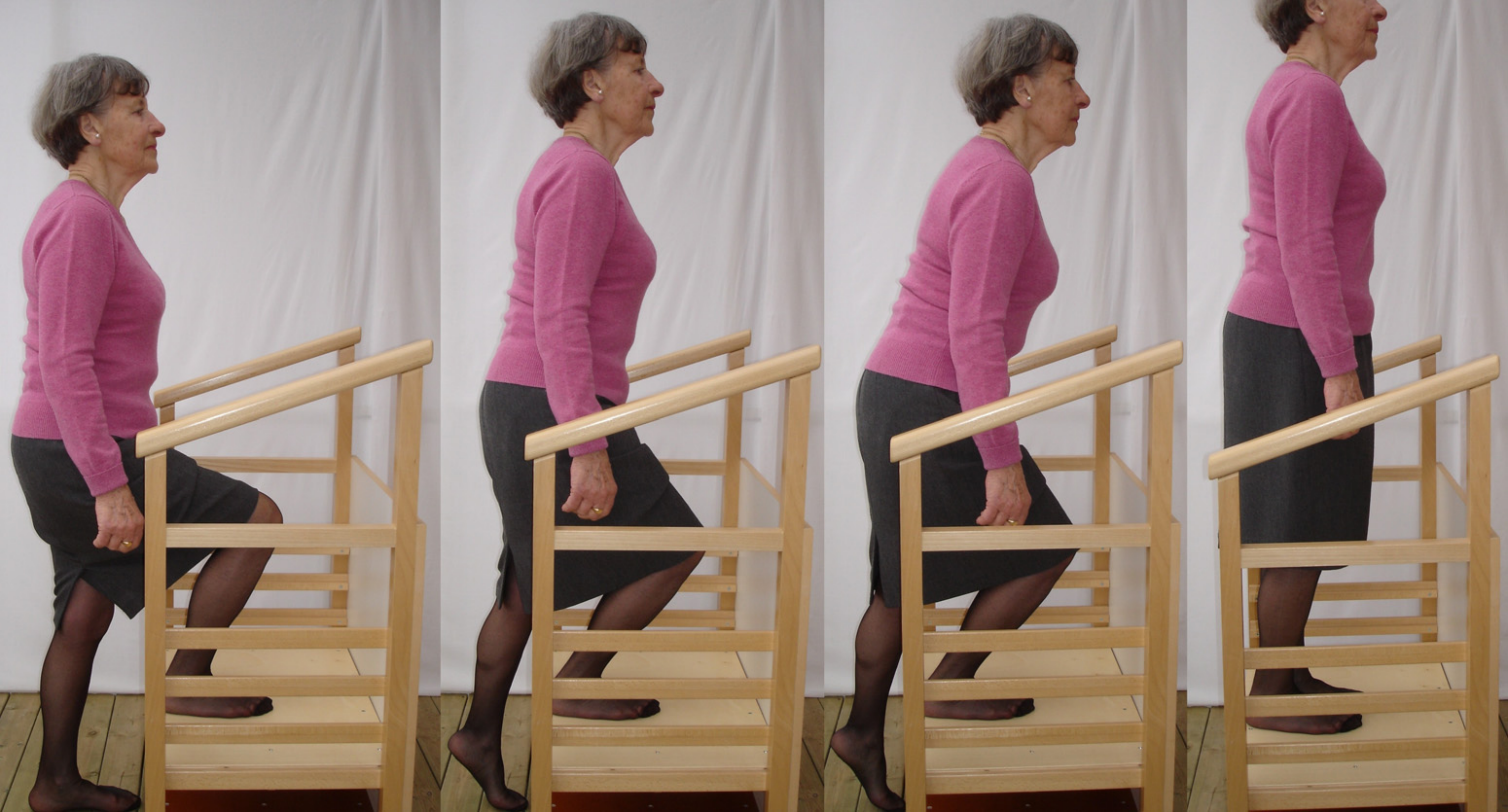
**The standardised maximal step-up test (MST)-instructions to subject**. First picture: after start position seen in the first picture, "go up as high as possible on the floor foot, stand still and find your balance". Second picture: "look forward and straighten your back while moving your body weight over to the step-up leg, stand still and again find your balance" as seen in the second picture. Third picture: "start the step-up by pressing the step-up foot into the board while extending in your knee, and during a slight bending forward slowly step-up onto the board". Fourth picture: end position with both feet on the board. After approved step-up test try a higher level, the highest approved level is the maximal step-up height for each leg.

The MSH (cm) in both legs was assessed, in 3-cm increments, with the standardised MST method (see Appendix 1) in a special device built by the first author (the step-up box, SB) shown in Figure 1. IKEPT (N·m, 60°·sec^-1^), essentially reflecting maximal concentric quadriceps strength, was assessed in an isokinetic dynamometer (Biodex System III PRO, Biodex Medical System, New York, NY, USA). Body weight was measured in light clothing without shoes to the nearest 0.1 kg using an electronic balance (Seca model). Height was measured without shoes to the nearest 0.5 cm using a scale fixed to the wall. BMI was calculated according to standard practice (kg· m^-2^). A locally adapted version of the self-administered International Physical Activity Questionnaire (IPAQ) "the last 7 days short form questionnaire" [22], was used for assessing self-reported physical activity (min·wk^-1^). Energy expenditure (kcal·wk^-1^) was calculated according to the MET (Metabolic Equivalent) estimates [23]. Mean energy expenditure from reported vigorous and moderate activity, and strength training time over a two-week period, was used.

To assess self-reported health-related quality of life, the Swedish version of the SF-36 was used [24]. The subscales physical function (PF), bodily pain (BP) and general health (GH) shown in table 1, all with a strong correlation to age [25], were used to describe subjects and to compare them with the general Swedish population. The values in the subscales range from 0 to 100. Values < 100 imply restrictions in function. The PF subscale items 3a-3j, and our selection of items 3a, b, d, f and g (table 2) assumed by the authors in this study to be suitable for assessment of healthy subjects, were used for comparison with the first assessment of MSH (mean of right and left leg) at occ 1. This scale was also used to investigate whether any MSH score cut-off level identified subjects with any limitation (score < 100) in physical function.

**Table 2:** Frequency of subjects with various degrees of limitation in physical function according to the items in SF-36, i.e., yes, limited a lot; yes, limited a little; no, not limited at all

SF-36, Physical Function	Severely limited	Somewhat limited	Not limited
Subgroup Item	Total (Women/Men)	Total (Women/Men)	Total (Women/Men)
	n = 60 (30/30)	n = 60 (30/30)	n = 60 (30/30)
3a vigorous activities	7 (6/1)	22 (10/12)	31 (14/17)
3b moderate activities	0 (0/0)	6 (5/1)	54 (25/29)
3d climbing several flights of stairs	0 (0/0)	3 (2/1)	57 (28/29)
3f bending, kneeling, or stooping	0 (0/0)	13 (8/5)	47 (22/25)
3g walking more than 2 km	0 (0/0)	2 (2/0)	58 (28/30)

### Testing procedures

The MSH was assessed with the standardised MST in the step-up device as described in Figure 1. The information provided to the examiner including how to instruct and encourage the subject performing the standardised MST is presented in detail in Appendix 1. The step-up test was performed in ordinary clothes, with bare feet or with socks. To avoid undesirable side effects of the MST, an incremental height increase was recommended. After the step-up demonstration the examiner asked if the subject had any current or previous problems with leg function, joint pain, and instability or muscle weakness. A low step height was selected for familiarisation with the test procedure. The tester supervised and approved the MST on one or both legs and the subject was then told to try a higher level. Three attempts at the highest level for each leg were allowed. The subjects were given verbal encouragement to perform at their best.

The third test occasion began with a MST of each leg followed by twenty minutes of seated rest, during which the physiotherapist described the IKEPT test. The subject had a 5-min warm-up on a bicycle before being placed in a sitting position in the Biodex device with a 90° flexion in the knee and hip. The manufacturer's standard test protocol was followed. An angular velocity of 60°·sec^-1 ^was chosen because it has been estimated to approximate that used when climbing ordinary stairs [26], and would not exhaust the subject. The subjects were given verbal encouragement and visual feedback to perform at their best.

### Statistics

Primary outcome variable for this study was MSH where the mean of right and left leg was used. Data from the first and the second, and from the first and the third test occasions, were analysed using a repeated measures ANOVA to estimate the within-subject standard deviation for further calculation of the test-retest repeatability, which is 2.77 times the within-subject standard deviation [27]. We expect 95% of differences between paired observations, i.e. test occasions, to be less than this definition of a repeatability adopted by the British Standards Institution [27,28]. MST at occ 3 was used to assess the relation to demographic factors such as sex, age, weight, body height, and BMI using multiple linear regressions. The validity of the MSH was investigated by analysing the relation between MSH at occ 3 and mean IKEPT of the right and left leg, using the Pearson correlation coefficient. The MSH at occ 1 was correlated to the SF-36 subscales using the Pearson correlation coefficient. Logistic regression was used to determine whether MSH could discriminate a cut-off score for any limitation (numerically < 100) or no limitation (= 100) in SF-36, PF. A cut-off level was estimated where the odds ratio for any limitation was set to 1.0. Subsequently, the cut-off level observed was used to compare predicted values against observed values. The measures of sensitivity, specificity and likelihood ratio were calculated to address the potential for MSH to discriminate subjects with any limitation in SF-36, PF. All tests were two-sided and P < 0.05 was regarded as statistically significant. Statistica, v8, StatSoft Inc, Tulsa, OK, USA, was used for statistical calculations.

## Results

### Characteristics of the study population

The characteristics of the subjects and scores on the SF-36 subscales are presented in table 1. The mean BMI indicates that both women and men were overweight. Energy expenditure data indicate moderate physical activity among men and less among women. Both sexes included subjects ranging from inactive to moderate active, who match patients likely to present in primary care for referral for physical activity on prescription [29], and to elite trained and consequently with a wide range of calculated energy expenditure. Assessment of SF-36 showed that our subjects had marginally better PF and BP, and about the same GH as the general Swedish population [25]. In our study 58.3% (34-64 years) had at least one limitation in PF, compared with 60.6% (35-64 years) in the general Swedish population [25]. Our subjects thus appear representative for the middle-aged general population in Sweden. The numbers of limitations observed in the domain of PF are presented in table 2. Nearly all limitations reported by our subjects were found in items 3a, b, d, f and g. Older subjects and women reported more limitations. The MSH range was 12-45 cm for women and 18-45 cm for men. The mean (SD) maximal step-up heights (cm) for each leg for all assessments at occasions 1, 2 and 3 are presented in table 3. The differences between legs and assessments were minor.

**Table 3:** Descriptive statistics using mean and SD for the maximal step-up height (MSH) in subjects at occasion 1 to 3

		Total, n = 60		Women, n = 30		Men, n = 30	
Occasion	Leg	Right	Left	Right	Left	Right	Left
		Mean (SD)	Mean (SD)	Mean (SD)	Mean (SD)	Mean (SD)	Mean (SD)
Occasion 1							
	First assessment	31.3 (5.8)	31.2 (4.9)	28.1 (4.9)	28.7 (4.2)	34.4 (4.9)	33.6 (4.3)
	Second assessment	31.7 (5.8)	31.5 (5.0)	28.4 (4.8)	28.7 (4.1)	35.0 (4.7)	34.2 (4.4)
Occasion 2							
	First assessment	32.2 (5.3)	31.8 (4.9)	29.0 (5.1)	29.0 (4.6)	35.3 (3.3)	34.4 (3.7)
	Second assessment	31.7 (5.9)	31.3 (5.3)	27.9 (5.4)	27.9 (4.4)	35.3 (3.8)	34.6 (3.9)
Occasion 3							
	Assessment	32.1 (5.4)	32.1 (5.0)	28.9 (4.4)	28.8 (3.9)	35.3 (4.2)	35.3 (3.6)

### Repeatability

The intra-examiner test-retest (one week between occasions) showed MSH repeatability of 6.9 and 5.9 cm for right and left leg, respectively. When four weeks passed between the first and third occasion the corresponding figures were 5.0 and 4.9 cm. The inter-examiner (minimum 30 minutes) repeatability was 9.6 and 8.5 cm (occ 1), and 6.6 and 5.6 cm (occ 2), for the right and the left leg, respectively.

### Correlations

**Figure 2:**
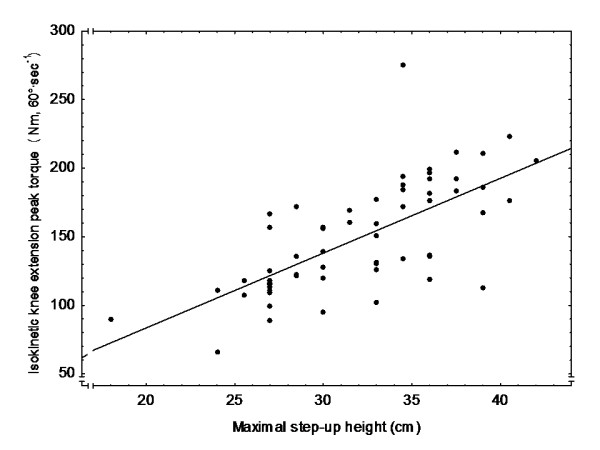
**Correlation between maximal step-up height and quadriceps strength**. Relation between the maximal step-up height (MSH, cm) and the quadriceps strength (isokinetic knee extension peak torque, IKEPT, N·m, 60°·sec^-1^) at occasion 3. Values for step-up height and quadriceps strength are calculated as the mean of the right and left leg.

MSH was correlated to IKEPT (*r *= 0.68, *P *< 0.001) (Figure 2), to SF-36, PF score (*r *= 0.29, *P *= 0.03) and limitations in the selected items in PF (3a, b, d, f, g) (*r *= -0.30, *P *= 0.02). MSH was not correlated to BP (*r *= 0.08, *P *= 0.53) and GH (*r *= 0.02, *P *= 0.88) scores of the SF-36.

### Regression analysis

The correlation of MSH to subject characteristics and self-reported physical activity is presented in table 4. Both models revealed significant inverse correlations. The first model revealed significantly lower MSH among women, and lower MSH with increasing age and BMI. The second model showed significantly lower MSH among women, and with increasing age and higher weight. Body height and self-reported physical activity did not significantly correlate to MSH. Results from the logistic regression modelling showed that MSH at the first test occasion could discriminate limitations in physical functioning, OR = 1.13, *P *= 0.037, and a corresponding cut-off level at 34 cm. The model had a sensitivity of 71% and a specificity of 58%. Subgroup calculations showed a cut-off level of 32 cm for women and 35 cm for men which discriminated those reporting any limitation in SF-36, PF. There were 7 out of 30 women and 14 out of 30 men having MSH > 32 cm and > 35 cm, respectively, on both legs.

**Table 4:** Results of regression coefficient and its corresponding standard error, *P*-value and R^2^, for variables correlated to the outcome variable MSH, after using multiple linear regressions

	Estimate B (SE)	Estimate of standardised B (SE)	*P*-value	R^2^
Model 1				0.598
Intercept	54.46 (4.07)		< 0.001	
Sex (Women = 1, Men = 0)	-7.34 (0.86)	-0.74 (0.09)	< 0.001	
Age (years)	-0.16 (0.07)	-0.22 (0.09)	0.020	
Body mass index, BMI, (kg·m^-2^)	-0.38 (0.11)	-0.31 (0.09)	0.001	
Model 2				0.594
Intercept	40.77 (16.35)		0.016	
Sex (Women = 1, Men = 0)	-7.76 (1.41)	-0.78 (0.15)	< 0.001	
Age (years)	-0.17 (0.07)	-0.22 (0.09)	0.020	
Height (cm)	0.08 (0.09)	0.13 (0.14)	0.341	
Weight (kg)	-0.13 (0.04)	-0.35 (0.11)	0.002	
Mean energy expenditure (kcal·wk^-1^)	0.00002 (0.0004)	0.0037 (0.09)	0.967	

Two different models are presented, using two different measures for body composition, model 1 uses BMI, and model 2 uses height and weight.

Leg problems and injuries (> 6 months before study) reported by the subjects could explain lower MSH and IKEPT values as well as low values in PF scores. The subjects did not report any adverse effects of the study and there were no dropouts. Performing the MST took 5-15 minutes and IKEPT took 25-30 minutes.

## Discussion

This study shows that the MST is a robust, safe and relevant test for leg strength and leg function in a healthy middle-aged population. Compared to IKEPT we rated MST as a fast test. The subjects limitations in physical function, their low to moderate energy expenditure and mean BMI > 25, indicated that many of the subjects in our study would be suitable candidates for a physical activity prescription when they present themselves in primary health care.

The measure of repeatability for this standardised MST indicates that 95% of all differences between occasions should be less than 6 cm. Larger differences are to be considered as "true", reliable individual changes. If MSH was assessed with increments of 1 cm the precision would probably be even better. Further, systematic changes on a group level, i.e. differences between or within groups, may be detected at a much lower level than 6 cm.

A few subjects of either sex in our study managed to step up to a high level (39-45 cm). Their quadriceps strength assessed by IKEPT was high and they had lower BMI, good joint mobility, coordination and balance, and reported no or tolerable pain. At this highest level the femur was parallel to the floor, and the knee angle was about 90° at the starting position for the step-up leg. For these subjects, this is probably the highest theoretical individual MSH with our standardisation.

The lower inter-tester difference between MSH at occasion 2 compared to occasion 1 could reflect a tester and/or subject learning effect. The threshold for maximum expected intra- and inter-tester differences in MSH in our study was 6 cm, which is less than the 10-cm interval used by other researchers when correlating step height to significant changes in leg strength and function [16,17,26]. Future studies are needed to further investigate the clinical significance of the 6 cm repeatability.

While not intending to predict knee-extension peak torque from MSH values, this study showed a significant correlation between MSH and IKEPT. This study indicates that MST is a valid test for knee extension function and less time-consuming. IKEPT values, used in our study as the gold standard, are highly reliable, and no learning effect has been observed [30]. Earlier results in two studies investigating the correlation between the maximum rising strength from sitting and isometric knee extension strength show correlations comparable to those in the present study [31,32]. Furthermore, MST assesses leg strength and performance in functional positions and movements and does not require expensive equipment as does IKEPT measurements.

We also found a significant correlation between MSH and SF-36, PF. Therefore, the standardised MST could be useful both to identify consequences of sarcopenia [33] and to support follow-up of treatment. Further studies are needed to investigate the effects of prescribed physical activity [34] on an individual's MSH, most likely corresponding to changes in knee extension function and self-reported physical function. In studies with older community dwelling populations objective measures of physical capability are predictors of all cause mortality [35].

On speculation, we explored whether a MSH cut-off value could identify perceived limitation of physical function and found cut-off values of >32 cm (women) and >35 cm (men) on group level. This might be of use for clinical assessment of the risk of falling or loss of functional independence in the future. Studies on larger groups of patients and comparisons with healthy subjects are needed before any further conclusions can be made.

We present MSH correlations to sex, age and demographics in two models with multiple linear regressions (table 4) indicating that the MST can be a useful tool for lifestyle interventions. In model 1, when BMI was used in the same model as weight and height were separately included, no correlations could be detected for height. The lack of correlation between MSH and body height in model 2, also found by other researchers investigating step height [15], could be due to the relatively small size of the study. The MSH did not correlate to self-reported physical activity. Objective estimates of physical activity [36] might have yielded lower values and a different activity pattern, possibly with lower variability. If so, a correlation between MSH and physical activity might have been identified.

## Conclusions

In clinical practice, objective measurements of physical function are seldom used and routines are generally not in place. The MST-assessing MSH on each leg-represents one aspect of current individual leg function, and is most likely dependent on leg muscle strength, but also on mobility, coordination, joint stability, balance and the degree of pain.

In conclusion, the maximal step-up test is simple to conduct, requires little equipment and space and can be performed by subjects in everyday clothing. We suggest that the maximal step-up test could be a useful and valid test of leg muscle strength and physical function and could be integrated into ordinary clinical routines.

## Competing interests

LAN have a patent relating to the device used for the step-up height assessments described in the manuscript, but have no financial relationship with any company or organisation. JK works as an independent consultant in biostatistics. He declares no conflict of interests. PA works as a physiotherapist. He declares no conflict of interests. The other authors declare that they have no competing interests.

## Authors' contributions

LAN developed and standardised the maximal step-up test, designed the study, participated in the acquisition of data, statistical analysis and interpretation of data, drafted and revised the manuscript. CJS, MLH and PW participated in the design of the study, analysis and interpretation of data, and in the drafting and revision of the manuscript. JK participated in the design of the study, performed the statistical analysis and interpretation of data, and in the drafting and revising the manuscript. PA participated in design, acquisition of data, interpretation of data and revising the manuscript. All authors have read the manuscript and agreed to its content.

## Appendix 1

**Title: **The standardised maximal step-up test (MST) assessing maximal step-up height (MSH)

**Description: **Detailed instructions to the examiner and to the subject

**I**. Begin with your demonstration of the MST at a low level, i.e. corresponding to an ordinary stair height. The step-up is done in ordinary cloths, with bare feet or with socks. To avoid any undesirable side effects an incremental increase of heights is recommended.

**II**. During the step-up demonstration, carefully explain the rules for the approved MST:

i) the foot of the step-up leg should be put in a place where good balance is established

ii) the arms should hang vertically at the sides

iii) stand in an upright position

iv) move slowly while performing the step-up

v) the pelvis should remain in a central position during the step-up

vi) avoid kicking off from the floor with the floor foot

vii) tilting backward or forward, or bending forward with the face passing the vertical line from patella, is not allowed.

**III**. After the demonstration ask the subject if there are any current or previous problems with leg function, joint pain or muscle weakness. Select a level at a low step height for training, and start with what the subject considers the strongest or dominant leg, to obtain familiarity with the test procedure. Then choose a MST start level at which the subject feels certain of being able to perform the test procedure in full compliance with the instructions. The subject is asked to start the MSH assessment with the leg suspected to be the weakest. If not approved MST try a lower level as the start level.

**IV**. Examiners instructions to the subjects during the MST (figure 1):

First: i) at starting position put the floor foot 5-10 cm in front of the step-up device, and put the step-up foot onto the board, ii) finish starting position by going up as high as possible on the toes of the floor foot, stand still and find your balance.

Second: look straight ahead and straighten your back while moving your body weight over to the step-up leg on the board in the step-up device, stand still and again find your balance.

Third: start the step-up by pressing the step-up foot into the board while extending in your knee, and during a slight bending forward slowly step-up onto the board.

Fourth: slowly put the floor foot onto the board in the step-up device and the MST is finished.

**V**. The examiner supervises and approves the MST on one or both legs and the subject is then told to try a higher level. Three attempts at the highest level for each leg are allowed. The subjects are given verbal encouragement to perform at their best. The highest approved level is the maximal step-up height for each leg.

## Pre-publication history

The pre-publication history for this paper can be accessed here:

http://www.biomedcentral.com/1471-2474/12/191/prepub
